# Cerebral salt-wasting syndrome due to hemorrhagic brain infarction: a case report

**DOI:** 10.1186/1752-1947-8-259

**Published:** 2014-07-23

**Authors:** Tomotaka Tanaka, Hisakazu Uno, Kotaro Miyashita, Kazuyuki Nagatsuka

**Affiliations:** 1 Division of Neurology, Department of Stroke and Cerebrovascular Diseases, National Cerebral and Cardiovascular Center, 5-7-1 Fujishiro-dai, Suita, Osaka 565-8565, Japan; 2 Department of Internal Medicine, Takarazuka Sanda Hospital, 2-22-10 Nishiyama, Sanda, Hyogo 669-1537, Japan

**Keywords:** Cerebral salt-wasting syndrome, Hemorrhagic brain infarction, Hyponatremia

## Abstract

**Introduction:**

Cerebral salt-wasting syndrome is a condition featuring hyponatremia and dehydration caused by head injury, operation on the brain, subarachnoid hemorrhage, brain tumor and so on. However, there are a few reports of cerebral salt-wasting syndrome caused by cerebral infarction. We describe a patient with cerebral infarction who developed cerebral salt-wasting syndrome in the course of hemorrhagic transformation.

**Case presentation:**

A 79-year-old Japanese woman with hypertension and arrhythmia was admitted to our hospital for mild consciousness disturbance, conjugate deviation to right, left unilateral spatial neglect and left hemiparesis. Magnetic resonance imaging revealed a broad ischemic change in right middle cerebral arterial territory. She was diagnosed as cardiogenic cerebral embolism because atrial fibrillation was detected on electrocardiogram on admission. She showed hyponatremia accompanied by polyuria complicated at the same time with the development of hemorrhagic transformation on day 14 after admission. Based on her hypovolemic hyponatremia, she was evaluated as not having syndrome of inappropriate secretion of antidiuretic hormone but cerebral salt-wasting syndrome. She fortunately recovered with proper fluid replacement and electrolyte management.

**Conclusions:**

This is a rare case of cerebral infarction and cerebral salt-wasting syndrome in the course of hemorrhagic transformation. It may be difficult to distinguish cerebral salt-wasting syndrome from syndrome of inappropriate antidiuretic hormone, however, an accurate assessment is needed to reveal the diagnosis of cerebral salt-wasting syndrome because the recommended fluid management is opposite in the two conditions.

## Introduction

It is not rare to encounter hyponatremia during the course of acute central nervous system (CNS) diseases. In the literature, hyponatremia complicates about 30% of subarachnoid hemorrhage cases [1,2] and 16.8% of head injury cases [3]. Syndrome of inappropriate secretion of antidiuretic hormone (SIADH) and cerebral salt-wasting syndrome (CSWS) have been reported as the major causes of hyponatremia, while the therapeutic policy is essentially opposite because of a difference in the circulatory blood volume. Therefore, it is crucial to differentiate SIADH and CSWS from the clinical viewpoint. There have been many reports of CSWS following subarachnoid hemorrhage, but CSWS accompanying ischemic cerebrovascular diseases has been rarely reported. We report the case of a woman with cerebral infarction who developed CSWS in the course of hemorrhagic transformation.

## Case presentation

A 79-year-old Japanese woman with a past medical history of hypertension and arrhythmia suddenly developed left hemiparesis and drowsy state, and was sent to our emergency room. A neurological examination showed mild consciousness disturbance, conjugate deviation to right, mild dysarthria, left unilateral spatial neglect, left facial and motor weakness, and left sensory disturbance. Magnetic resonance imaging depicted a large high intensity lesion in right middle and posterior cerebral arterial territory on diffusion weighted images, and her right internal carotid artery was obstructed on magnetic resonance arteriography (MRA). Atrial fibrillation was observed on electrocardiogram. No other vascular lesion which may have caused the disease was noted, suggesting cardiogenic cerebral embolism. Her blood analysis showed almost normal findings including hemoglobin 13.0g/dL, hematocrit 39.8%, sodium (Na) 142mEq/L, blood urea nitrogen (BUN) 16mg/dL, and creatinine, 0.69mg/dL. In addition to anti-edema therapy, the continuous intravenous infusion of heparin was initiated on the following day because no apparent hemorrhage was identified on computed tomography (CT). No marked change was noted in neurological signs, and her brain edema tended to improve on CT. On day 14, her right internal carotid artery became patent on MRA, and hemorrhage occurred in the infarct region of her right middle cerebral artery. Thus, heparin administration was discontinued on the same day.

**Figure 1:**
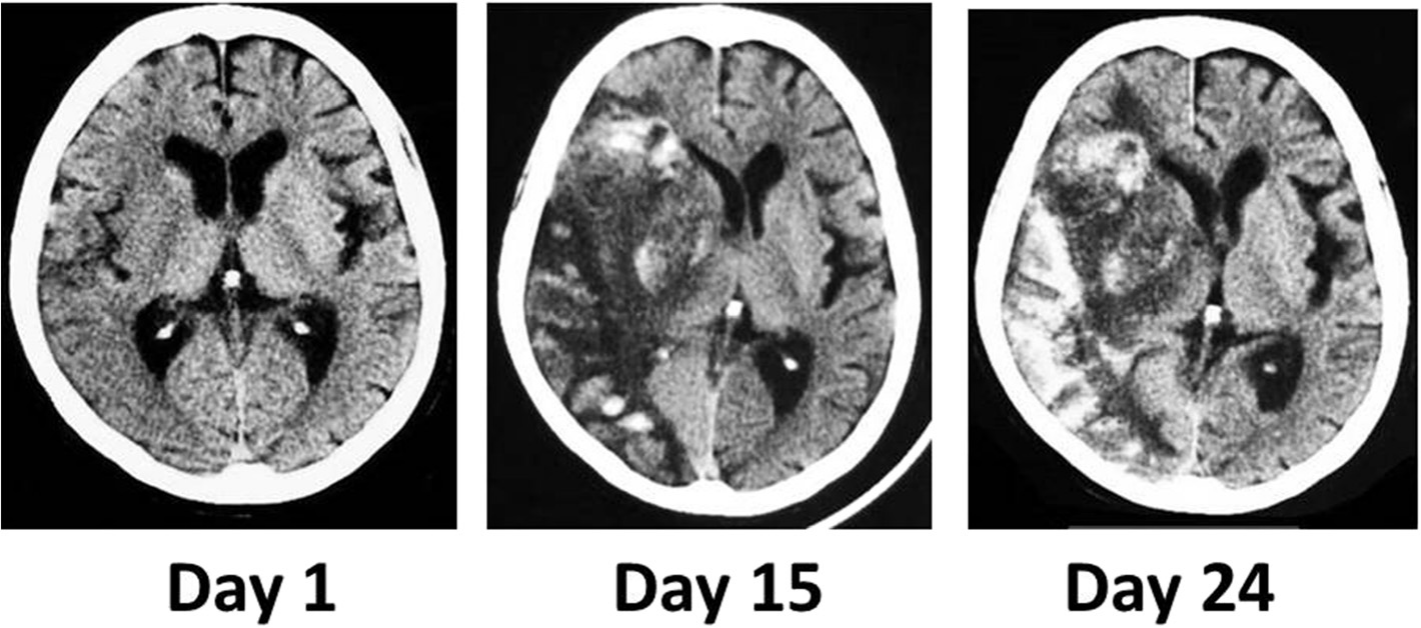
**Changes of cerebral infarction in computed tomography.** Slight, broad low density area in the right middle cerebral artery territory was depicted on day 1, showing the early cerebral infarction. Large low density area with midline shift and high density was depicted on day 15 and day 24, showing hemorrhagic transformation and severe brain edema.

**Figure 2:**
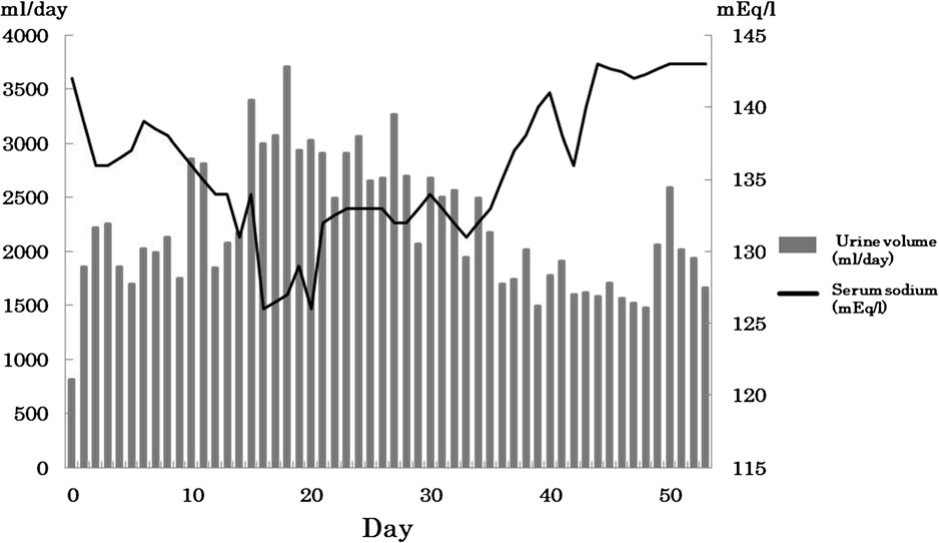
**Time course of serum sodium value and urine volume.** Two weeks after admission, hyponatremia and diuresis progressed following hemorrhagic transformation. The urine volume and sodium value were normalized with precise fluid and sodium correction and improvement of hemorrhagic change and edema.

However, hemorrhage and brain edema expanded on day 15 (Figure 1), and consciousness disturbance deteriorated. Concurrently, in addition to polyuria and hyponatremia (Figure 2 and Table 1), features of dehydration appeared, such as reduction of skin turgor, collapse of the inferior vena cava (IVC), and weight loss. Fluid replacement induced only an increase of urine volume and failed to correct dehydration tendency (input of 3410mL compared to a total urinary output of 3710mL on day 20). Considering the possibility of diabetes insipidus, a water deprivation antidiuretic hormone stimulation test (by inserting 10mcg of desmopressin into a nostril by using the spray once) was performed on day 28, however, her urine volume did not decrease. Despite adequate fluid replacement, she was in negative fluid balance and her weight had decreased by 4.6%, from 39.2kg to 37.4kg. An endocrinological examination excluded SIADH because of hydration features. CSWS was assumed to be a more probable pathological state, and salt supply was added to fluid replacement on day 29. Following the alleviation of hemorrhagic transformation, her excessive urine volume slowly decreased, and her hyponatremia and dehydration improved. She was transferred to another rehabilitation hospital about 2 months after admission.

**Table 1:** The transition of biochemical parameters related to the abnormality of sodium metabolism in the patient

	**Days from onset**		
**Biochemical data**	**Day 15**	**Day 25**	**Day 52**
Serum sodium (mEq/L)	134	133	143
Uosm (Osm/kg·H2O)	454	385	266
Posm (Osm/kg·H2O)	279	no data	no data
Urinary sodium excretion (mEq/day)	219	239	179
FEUA (%)	26	16	12

FEUA, fractional excretion of uric acid; Posm, plasma osmolality; Uosm, urine osmolality.

## Discussion

CSWS was reported as a condition of urine volume increase and excess loss of urinary Na accompanied by weight loss in intracranial hemorrhagic disorders [4]. Hyponatremia is a common complication in CNS diseases, especially head injury and cerebrovascular disorder, and has been regarded as SIADH in general. Considering dehydration associated with hyponatremia, however, CSWS was more common than SIADH in CNS diseases as Nelson *et al.* pointed out [5]. Since then, many cases of CSWS have been reported, while the pathogenesis of CSWS has remained unexplained. Some reports showed that atrial and brain natriuretic peptides (ANP and BNP) from damaged CNS tissues and abnormality of the sympathetic nervous system might induce CSWS [6-9]. That is, the increasing natriuretic peptide secretion and catecholamine might cause natriuresis via reduced renin secretion [9-12]. In our case, the level of ANP and BNP continued to be elevated (BNP 250.2pg/mL, ANP 200pg/mL) after the improvement of cardiac function.

CSWS is frequently associated with CNS hemorrhagic diseases, but only a few cases of CSWS in brain infarction have been reported [11-13]. The previous case reports of cerebral infarction had the characteristics of severe physical stress including systemic autoimmune disease, serious infection, and trauma (Table 2) [11,13]. These systemic complications might be more susceptible to developing CSWS than localized involvement in patients with cerebral infarction. In fact, in our patient, manifestation of CSWS appeared not in the early stage but in the advanced stage with hemorrhagic transformation and excessive edema of cardiogenic brain infarction.

**Table 2:** Patients with cerebral salt-wasting syndrome accompanying cerebral infarction

**Sex/age**	**Cerebral infarction type**	**Lesion**	**Excessive edema**	**Hemorrhagic transformation**	**Comorbidity**	**Days from onset (days)**	**Treatment**	**Serum sodium concentration (mmol/L)**	**Urine volume**	**Uosm (mL/day)**	**Posm (mL/day)**	**Ref. no.**
M/3	Dissection of ICA	Right MCA (broad)	Yes	No	Head injury	6	Intravenous saline	122	4.3mL/kg/hour (BW: 17.0–17.5kg)	607	219	[11]
F/38	Unknown	Right MCA (broad)	Yes	no data	Systemic lupus erythematosus	5	Intravenous saline	112	2800mL/day (BW: no data)	280	276	[13]
					Lupus nephritis		Hydrocortisone					
					Tuberculous meningoencephalitis							
F/79	Cardiac embolism	Right	Yes	Yes	Atrial fibrillation	15	Intravenous saline	134	3710mL/day (BW: 37.4–39.2kg)	454	279	Current report
		MCA (broad)			Hypertension		Oral salt					

BW, body weight; F, female; ICA, internal carotid artery; M, male; MCA, middle cerebral artery; Posm, plasma osmolality; Uosm, urine osmolality.

Whereas SIADH management requires strict fluid volume restriction, the treatment policy of CSWS is adequate fluid balance control and substitution of sodium chloride. Distinguishing these conditions may be unexpectedly difficult [14] because they show much in common; they have overlapping clinical and biochemical signs such as absence of peripheral edema, normal renal and adrenal function, low serum Na, low serum osmolality, and high urinary Na. Glycerol and mannitol as hyperosmolar agents are commonly used for brain edema, making it difficult to assess the true urinary and plasma volume. Extracellular volume assessment is a key factor to distinguish CSWS from SIADH. Due to a lack of precise assessment of the amount of extracellular fluid, it is necessary to note clinical dehydration signs such as reduction of turgor, raised heartbeat, body weight loss and collapsed IVC as well as laboratory findings including BUN/creatinine ratio. Furthermore, the persistence of hypouricemia and elevation of fractional excretion of uric acid (FEUA) even after the correction of hyponatremia may contribute to differentiate CSWS from SIADH as already reported [15]. In our patient, the serum Na level normalized on day 52, but the FEUA was still 10% higher than normal range (Table 1).

## Conclusions

We reported a case of CSWS following cerebral infarction. In patients with severe stroke, disturbances of Na metabolism are frequent. It is important to investigate the causes of hyponatremia in patients with stroke, considering the differential diagnosis of SIADH and CSWS by assessing the dehydration signs.

## Consent

Written informed consent was obtained from the patient for publication of this case report and accompanying images. A copy of the written consent is available for review by the Editor-in-Chief of this journal.

## Abbreviations

ANP: Atrial natriuretic peptides; BNP: Brain natriuretic peptides; BUN: Blood urea nitrogen; CNS: Central nervous system; CSWS: Cerebral salt-wasting syndrome; CT: Computed tomography; FEUA: Fractional excretion of uric acid; IVC: Inferior vena cava; MRA: Magnetic resonance arteriography; Na: Sodium; SIADH: Syndrome of inappropriate secretion of antidiuretic hormone.

## Competing interests

The authors declare that they have no competing interests.

## Authors’ contributions

TT wrote the manuscript. TT and HU acquired patient data. HU and KM reviewed the case notes and were major contributors in writing the manuscript KN edited the manuscript and provided suggestions. All authors read and approved the final manuscript.
